# Supplementary material for: Towards responsible surveillance in preventive health data-AI research
Source: PLOS Digit Health. 2025 Dec 29;4(12):e0001146. doi: 10.1371/journal.pdig.0001146 (PMC12747365; doi:10.1371/journal.pdig.0001146)
Supplement: Attachment — Submitted filename: Response to reviewers_responsible surveillance.docx [file pdig.0001146.s002.docx]

| Comment | Response | Changes (all markup) |
| --- | --- | --- |
| Reviewer #1 |  |  |
| The paper is coherent and well-structured. The authors effectively build their case by establishing the historical context of surveillance in medicine and then demonstrating how AI technologies amplify and transform these existing practices. In my opinion, the paper makes a significant contribution to the field by moving beyond a purely critical stance to offer a concrete and actionable framework for governance, thereby addressing a noted gap in the critical surveillance literature. Despite its considerable strengths, the paper’s argument is not without weaknesses and important nuances that must be considered. The most significant of these is its use of the Hypermarker project as its central case study. The analysis is selective, highlighting the project as an exemplar of surveillance risks while failing to engage with the project’s own explicit and documented commitments to ethical safeguards such as “privacy by design” and stakeholder co-creation. This omission makes the use of the case study appear more as a convenient illustration than as a balanced piece of evidence. | We thank the reviewer for their constructive and thoughtful appraisal of our paper. We agree that the paper would benefit from a more structured use of the Hypermarker project as a case study, particularly by engaging more explicitly with its ethical safeguards. In response, we have incorporated a more detailed discussion of the project’s safeguards and measures under ‘Ethical and social issues of surveillance in health AI research’ to provide a more balanced and substantiated account. | Changed and added text, p. 4 lines 145-152: We begin by introducing the Hypermarker research project as a case study of health data-AI research. We then trace the evolution of surveillance within medicine, public health, data-driven research, and the proliferation of digital health technologies, before examining how the development of AI technologies amplifies and transforms these existing practices. Subsequently, we analyse the implications and ethical and social issues associated with surveillance in health data-AI, and critically assess the safeguards and measures implemented in the Hypermarker project. We conclude by identifying key challenges and providing recommendations for advancing responsible surveillance practices in health data-AI research.  Added text, p. 11, lines 317-334: Building on this characterization of health data-AI surveillance, we examine its most important implications as well as the associated ethical and social issues. In light of these, we critically assess the safeguards and measures implemented in the Hypermarker project. These can be grouped into three main categories:  1. Technical procedures, including federated data analysis and the Findable, Accessible, Interoperable, and Reusable (FAIR) harmonisation of data drawing upon the International Council for Harmonisation of Technical Requirements for Pharmaceuticals for Human Use’s Guideline for Good Clinical Practice (ICH-GCP) [37].  2. Ethical and regulatory safeguards, focusing on ethics- and privacy-by-design principles, as well as data protection and privacy frameworks informed by the European Union’s General Data Protection Regulation (GDPR, EU 2016/679) and Medical Device Regulation (MDR, 2017/745), the World Medical Association (WMA)’s Declaration of Helsinki [38], and the European Commission’s Independent High-Level Expert Group on AI’s Ethics Guidelines for Trustworthy AI [39] and Assessment List for Trustworthy AI (ALTAI) [40].  3. Patient and public involvement and engagement (PPIE), particularly in clinical trial design, health economics, and responsible governance.  Added text, pp. 13-14, lines 374-408: The project addresses the implications of AI’s pervasiveness and unobtrusiveness for transparency, accountability, and personal autonomy. Transparency is primarily operationalised through traceability and explainability measures. In selecting AI models, the project explicitly considers the trade-off between interpretability and accuracy, prioritising interpretable models such as LASSO (Least Absolute Shrinkage and Selection Operator) and Decision Trees. More complex models are adopted only when justified by substantially improved performance. In such cases, extensive screening is conducted using explainable AI (XAI) tools, such as feature importance measures and both model-dependent and model-agnostic methods, to ensure a clear understanding of how predictions are generated. Dedicated methods are also employed to elucidate the reasoning behind more complex, so-called ‘black-box’ models. These explanations are made accessible to end-users in plain language and integrated into the decision-support tool’s outputs. Beyond explainability, the project emphasises transparency by adhering to established reporting standards from EQUATOR Network initiatives, including TRIPOD [42], DECIDE-AI [43], and STARD [44], which promote rigorous and transparent health AI research.  Accountability is addressed only to a limited extent, primarily through measures focused on auditability and risk management. The project promotes accountability by ensuring that code is made publicly available in open repositories, and that training data are accessible via the Hypermarker Data Catalogue and Hypermarker Federated Data Repository. These infrastructures are implemented in accordance with principles of open science and privacy-by-design, supporting transparency, reproducibility, and responsible data stewardship.  Autonomy is primarily addressed under the principle of human agency and oversight. Hypermarker designs its modelling processes to guide and support, rather than replace, human decision-making. The final predictive algorithm will be implemented as a clinical decision-support tool, accessible through a dashboard serving as the user interface for both clinicians and patients. Within this dashboard, it will be made explicitly clear that the prediction results derive from an algorithmic process. Predictions will be presented in accessible, lay terms, accompanied by detailed explanations of how they were generated. These explanations build on the project’s explainable AI analyses and comply with EU in vitro diagnostic medical device regulations. Confidence intervals will be displayed and associated risks highlighted to mitigate overreliance on the system. The model functions solely as a decision-support mechanism, with final treatment decisions remaining entirely the responsibility of the clinician (human-in-command). To avoid potential confusion, no human-like simulation will be developed for end users. Instead, targeted training will be provided to ensure appropriate and informed use of the tool.  Added text, pp. 15-16, lines 440-456: The project addresses privacy challenges arising from hypercollection and function creep by focusing on privacy standards, data quality and integrity, secure access, and stakeholder participation. While trust is not explicitly addressed, privacy protection is operationalised through multiple technical and procedural safeguards. All data collection and analytical activities are conducted in compliance with recognised privacy standards to ensure the quality and integrity of data. Data usage policies are communicated to users, and informed consent is obtained for the collection and processing of personal information. In addition, robust procedures are implemented to secure data access in accordance with GDPR principles. For the final decision-support tool, the FAIR Digital Twinning approach incorporates encryption at the source and a multi-level privacy protection architecture. Only the subsets of data necessary for a specific research question are used, in alignment with the user’s consent. A digital ‘twin’ of a real-world individual is created for analysis, while end-users can access only the analytical outcomes within a safe, secure, and FAIR environment, promoting the ethical use and responsible reuse of patient data. Patients, clinicians, and other relevant stakeholders are involved in the design and development of the clinical decision-support tool. Through co-creation, the project aims to embed values such as privacy into the resulting technologies and governance processes.  Added text, p. 17, lines 494-507: The Hypermarker project addresses the impact of profiling and hypervisibility, manifested through bias, unfairness, inequities, and potential stigmatisation, by emphasising diversity, non-discrimination, mitigation of unfair bias, and stakeholder participation. Before addressing potential biases in AI modelling, inherited biases in data that underpin these models but may not fully represent population diversity are considered. To mitigate such effects, the project commits to transparent reporting and communication of findings, guided by established reporting standards such as STROBE [50]. Balanced sampling strategies and metadata considerations are employed to address potential biases in training data and to promote fairness across dimensions such as gender, ethnicity, and disability. Regarding gender, the derivation cohort is drawn from multiple countries with gender parity in sampling. Trial recruitment includes stratification of the primary outcome by gender, and algorithm development explicitly treats gender as a key covariate. Through participatory processes, the project aims to embed non-discrimination and service to the public good into the development and governance of health AI.  Added text, p. 18, lines 529-530: Beyond accountability (as addressed above), the effect of informational power on public trust and equity is not directly addressed in Hypermarker.  Added text, p. 19, lines 555-556: Beyond privacy, autonomy, and transparency (as discussed above), the surveillant assemblage’s effects are not addressed in the Hypermarker project. |
| It should be noted that the paper's heavy reliance on European scholarship and its grounding in a policy context shaped by regulations like the GDPR may limit the direct applicability of its specific governance recommendations in other legal and political environments, such as the more sector-specific privacy landscape of the United States. | We have broadened the scope of our analysis by elaborating on the implications of health AI surveillance, the associated ethical and social issues, and key challenges, while also clarifying how the Hypermarker project’s safeguards and measures are embedded within broader international guidance and standards. | Removed text, p. 20: We have distinguished the issues related to trust, equity, transparency, accountability, autonomy, and privacy. This bears strong resemblance with, yet slightly deviates from, the principles mentioned in vested guidance for responsible AI, such as the Ethics Guidelines for Trustworthy AI [39]. As our focus is on the socio-ethical issues and implications of surveillance in the context of preventive health data and AI research, we have identified these principles and values as particularly relevant. While they align with those found in existing guidelines, they also extend and complement them. This is especially important in addressing the deeper socio-technical implications of surveillance in health AI research.  Changed text, p. 24, lines 548-551: These challenges underscore the importance of broad societal involvement in shaping the ethical frameworks and governance of health data-AI research. Regulatory initiatives have already laid important foundations by emphasizing transparency, accountability, and human oversight.  *See also changes made in response to Reviewer 1’s first comment.*  *See also changes made in response to Reviewer 2’s first comment.* |
| Reviewer #2 |  |  |
| Alright, here’s the thing. This paper takes on a big, timely, kinda scary topic—health surveillance in the age of AI. You do a nice job walking us through the history of surveillance and then setting the stage with today’s personalized medicine and hyper-data-collection mess. The bit about intentional vs unintentional surveillance? Super useful. And the way you point out issues like hypercollection, profiling, and power concentration—yeah, spot on. Also, kudos for the framework on trustworthiness, transparency, and collective oversight. That’s gold. But. (You knew there was a “but,” right?) This still reads more like a polished perspective piece than a research article. Which is fine—except you’ve slotted it under “Research Article.” So let’s talk tweaks:  1. Bring Hypermarker into the spotlight.  You dangle this fascinating Hypermarker project as a case study… and then barely touch it again. It sits mostly in a box like an afterthought. That’s a missed opportunity. The theoretical discussion is good, but readers need to see those concepts in action. So: when you talk about hypercollection and function creep, connect it directly—what happens if Hypermarker’s pharmacometabolomic data gets reused down the line? When you dig into profiling and hypervisibility, who exactly might be most at risk if Hypermarker’s outputs lean into known hypertension disparities? And those governance recommendations—what would collective oversight actually look like in this specific consortium? Pull Hypermarker through the narrative instead of leaving it parked on the side. | We thank the reviewer for appreciating and valuing the contributions of our paper. Building on the revisions made in response to Reviewer 1’s comment regarding a more explicit engagement with the Hypermarker project’s ethical safeguards, we have further elaborated the ethical and social issues drawing more explicitly on the Hypermarker project. We have. In line with these changes, we have also revised the abstract, author summary, and conclusion of the paper. | Changed text, pp. 1-2, lines 14-36: Abstract  The integration of artificial intelligence (AI) into health data research promises to transform precision medicine, especially by managing complex and chronic conditions like hypertension through decision support. Yet health AI also furthers surveillance, with serious ethical and social impact. Nevertheless, surveillance in health, in particular data-AI research and innovation, is understudied. This paper provides a conceptual analysis of health data-AI surveillance using the Hypermarker research project as a case study. We trace the evolution of surveillance within medicine, public health, data-driven research, and the proliferation of digital health technologies, before examining how the development of AI technologies amplifies and transforms these existing practices. We analyse health data-AI surveillance’s implications of pervasiveness and unobtrusiveness, hypercollection and function creep, hypervisibility and profiling, informational power, and the formation of a surveillant assemblage, followed by an assessment of the safeguards and measures implemented by the Hypermarker project. Our analysis exposes several key challenges for responsible surveillance practices in health data-AI research: strengthening trustworthiness through fairness and equity, ensuring accountability through transparency, and fostering public control and oversight. To this end, we recommend advancing responsible governance by implementing arrangements such as community advisory panels, independent review boards and oversight bodies, data-AI justice frameworks and dialogues, transparency dashboards and public AI portals, stewardship committees, accountability assemblies, and open oversight cycles  Changed text, p. 2, lines 40-49: Author Summary  In this study, we explore how the growing use of artificial intelligence in health research is changing the way information about people is collected, used, and governed. We focus on a case study that aims to improve treatment for high blood pressure and show how systems designed to support medical decisions can also create new forms of surveillance. This means that more and different kinds of information about people are gathered, often in ways they do not see or control. Such practices can affect privacy, fairness, and trust, and may deepen inequalities in healthcare. We show that these issues are not only technical or ethical, but also about how decisions are made and who is involved in making them. Building trustworthy and fair uses of artificial intelligence in health requires openness, accountability, and shared oversight. We suggest that patients, communities, and the public should have a stronger role in shaping how these technologies are developed and used.  Added text, pp. 12-13, lines 363-373: In Hypermarker, pharmacometabolomic profiling is designed to detect subtle biomarkers of hypertension across diverse populations. While this continuous monitoring improves early detection, it also normalises the constant observation of biological processes. Such unobtrusiveness risks obscuring the extent to which individuals are being monitored, potentially eroding awareness of data collection boundaries. This raises ethical questions about ongoing consent, data minimisation, and the extent to which patients and clinicians can meaningfully grasp the scope of AI-enabled observation embedded in everyday healthcare and research infrastructures.  Added text, p. 15, lines 432-439: Hypermarker exemplifies hypercollection, the aggregation of vast, heterogeneous datasets including biological samples, clinical trial data, and multi-omic information. While these data are crucial for training robust predictive models, their richness increases the likelihood of function creep, where data are repurposed for new analyses or commercial use beyond the project’s original intent. For instance, pharmacometabolomic data collected for hypertension research could later be used for unrelated biomarker discovery without renewed consent. The very structure that enables large-scale integration also amplifies the ethical tension between scientific value and data sovereignty.  Added text, p. 17, lines 487-493: Hypermarker’s predictive algorithms stratify patients according to risk of hypertension and treatment response, potentially reproducing or amplifying existing inequities. If training data underrepresent certain demographic or socioeconomic groups, the resulting models may produce skewed risk profiles that render already disadvantaged populations hypervisible as ‘high risk’. This could reinforce stigma or justify differential treatment pathways. For example, minority or low-income groups disproportionately affected by hypertension may face intensified monitoring or exclusion from tailored interventions.  Added text, p. 18, lines 522-528: The data infrastructures underpinning Hypermarker exemplify the informational power characteristic of contemporary surveillance. The capacity to collect, integrate, and infer from multi-modal health data positions AI systems as powerful epistemic agents, shaping not only medical knowledge but also the allocation of resources and the definition of health risks. In Hypermarker, predictive insights could influence clinical decisions or policy priorities, thus redistributing epistemic authority away from patients and practitioners toward algorithmic systems and data managers.  Added text, p. 19, lines 547-554: Hypermarker operates within a surveillant assemblage, a distributed network of actors, technologies, and institutions that collectively produce and manage health data. This assemblage includes hospitals, research institutions, data repositories, AI developers, and governance bodies. Each contributes to the continuous flow, analysis, and interpretation of personal health information. While this networked model enables collaborative innovation, it also diffuses accountability, since no single actor fully controls how data circulate or are repurposed. The complexity of distributed surveillance raises persistent questions about how to maintain ethical coherence across diverse sites of control and responsibility.  Revised text, pp. 28-29, lines 2499-2518: Conclusion  The surveillance induced by health data-AI research entails implications of pervasiveness and unobtrusiveness, hypercollection and function creep, hypervisibility and profiling, informational power, and the formation of a surveillant assemblage. While the safeguards and measures implemented by the Hypermarker project address many of the ethical and social issues arising from these dynamics, they also expose several persistent challenges for responsible surveillance practices in health data-AI research. Strengthening trustworthiness through fairness and equity, ensuring accountability through transparency, and fostering public control and oversight emerge as key governance challenges. To this end, mechanisms such as community advisory panels, independent review boards and oversight bodies, data-AI justice frameworks and dialogues, transparency dashboards and public AI portals, stewardship committees, accountability assemblies, and open oversight cycles play an essential role in advancing responsible surveillance practices in health data-AI research. |
| 2. Clarify the research “method.”  Right now, the structure is a bit confusing. You call it a research article, but it doesn’t walk or talk like one—there’s no Methods section. Readers will appreciate a short, no-frills “Methods” chunk right after the intro. Something like: this is a conceptual analysis using Hypermarker as an illustrative case, supported by literature from bioethics, surveillance studies, and health policy. Boom. Clear. | We have included a Materials and Methods section to clarify the paper’s conceptual analysis and case study methodology. | Added text, pp. 4-5, lines 153-177: Materials and Methods  This paper employs a conceptual approach, using the Hypermarker research project as an illustrative case study to explore the ethical and social dimensions of surveillance in health data-AI. The analysis integrates insights from bioethics and the social sciences, in particular surveillance and science and technology studies (STS), to critically examine how AI-driven innovations in health research reconfigure existing practices of data collection, monitoring, and governance.  The case study of Hypermarker, a health data research project developing an AI-driven decision support system for personalization of hypertension treatment, serves as an empirical reference point for situating ethical and social issues within an ongoing research context. Relevant project documentation, public materials, and governance frameworks were reviewed to identify the safeguards and measures implemented to promote responsible research practices.  The analysis proceeds through three stages. First, it traces the evolution of surveillance practices in medicine, public health, and data-driven research, highlighting continuities and transformations introduced by AI technologies. Second, it examines the implications of these developments, highlighting several crucial ethical and social issues such as privacy, trust, transparency, and accountability. Third, it critically assesses the Hypermarker project’s approach to responsible surveillance. Finally, it draws lessons for the development of ethically robust and socially responsive governance frameworks for surveillance in health data-AI research. |
| 3. Make the recommendations bite-sized and practical.  The trust/oversight/accountability trio is strong in theory, but at times it feels like you’re waving from 30,000 feet. Readers—especially policymakers and researchers—need to know: how does this translate on the ground? If you say “participatory governance structures,” don’t stop there. Spell out one or two models. Maybe a citizen advisory board. Maybe a data trust with teeth. Maybe structured co-design workshops where patients and communities actually get a voice. Concrete examples will make this much more actionable. | We have revised the key challenges and recommendations to enhance their tangibility and actionability.  We have moved the recommendations to a separate ‘Recommendations for governance of health data-AI surveillance’ section. | *See the revisions to ‘Key challenges for responsible surveillance practices in health AI research’ on pp. 20-25*  Changed text, pp. 26-28, lines 2295-2497: Recommendations for governance of health data-AI surveillance  Building on the relational understanding of trust, community advisory panels can help translate principles for trustworthiness into practice. To counteract hypercollection and function creep, such participatory structures enable sustained deliberation between researchers, developers, and community representatives, ensuring that public concerns shape decisions around data use, algorithmic design, and the visibility of data collection. For instance, research and innovation initiatives could employ community advisory panels to co-design transparency and communication strategies for AI diagnostic tools, demonstrating how trust can be built through visible and repeatable engagement.  Another way to operationalize reproducible trust is through independent review boards, such as ethics or equity boards, that conduct ongoing reviews of fairness and bias in AI systems. These boards can integrate algorithmic impact assessments throughout research lifecycles to monitor and address emerging equity risks, ensuring that trustworthiness remains a continuous and verifiable process rather than a static attribute. By doing so, they counteract the informational power held by data managers and commercial actors, redistributing epistemic authority back to patients and practitioners.  Profiling and hypervisibility require targeted governance mechanisms to mitigate bias, inequity, and stigmatization. Developers and researchers can adopt a data-AI justice framework that embeds fairness and inclusivity at the design stage. This involves systematic auditing of datasets for demographic bias, validation with affected groups, and transparent documentation of data provenance. In addition, initiatives could pilot data-AI dialogues with underrepresented communities to co-design metadata and ensure that local health realities are reflected in data infrastructures, and that data are not repurposed beyond the scope of consent.  Accessible transparency dashboards that report AI performance across demographic groups can sustain public confidence, making algorithmic risk stratifications and real-time AI monitoring visible and contestable, restoring participant awareness and agency in contexts where surveillance is otherwise invisible. Together, these measures help ensure that fairness and equity are not abstract aspirations but lived realities within evolving health AI systems. Transparency can be further strengthened through public AI portals, digital platforms that allow people to see what data are collected, how algorithms function, and who is accountable for their outcomes. In light of the unobtrusive and pervasive monitoring inherent in AI-driven health systems, such portals could explain the purpose, data sources, and decision logic behind AI systems in health services, provide simplified descriptions of AI models, publish regular performance audits, and include accessible channels for questions or complaints.  Translating accountability through transparency into practice requires visible and sustainable mechanisms of accountability to address. One effective approach is the establishment of stewardship committees comprising researchers, patient representatives, and civil society members who review data-sharing practices, secondary data use, and algorithmic development decisions across the network, ensuring coherence in a distributed system. These committees can conduct multi-stakeholder reviews of research proposals, ensuring that innovation proceeds within clearly defined and publicly legitimate boundaries.  Participatory models such as accountability assemblies or juries, which deliberate on the ethical and social implications of health data-AI surveillance, can also strengthen institutional accountability. These forums provide structured opportunities for communities and publics to question data practices, review audit results, and co-create principles for acceptable data use, thereby directly addressing the risks posed by stratified surveillance, fostering informed public influence over AI governance.  Public control on the surveillant assemblage, where data circulate across distributed actors and institutions, can be further institutionalized through independent oversight bodies that combine professional expertise and lived experience, moving beyond consultation towards co-decision-making and giving communities real influence over how data are used and algorithms validated in alignment with public values. To complement consent-based mechanisms and local review, wider citizen assemblies can be convened to deliberate key governance questions, such as data sharing with industry. By integrating public reasoning into technical decision-making, these assemblies enable publics to weigh societal trade-offs collectively and ensure that data and AI innovation evolves within democratically negotiated boundaries. In addition, open oversight cycles, consisting of periodic public reviews in which oversight bodies publish plain-language reports on how data are used, shared, and governed, can further reinforce accountability.  Ultimately, effective public control depends on layered, participatory structures that connect everyday governance with strategic oversight. Combining community advisory panels, independent review boards and oversight bodies, data-AI justice frameworks and dialogues, transparency dashboards and public AI portals, stewardship committees, accountability assemblies, and open oversight cycles enables public influence to operate across everyday data-AI practices, strategic oversight, and broader policy decisions. These structures ensure that trust, equity, and accountability are co-produced in practice, rather than imposed externally, and that the ethical challenges of pervasive, hypercollected, and algorithmically mediated health surveillance are directly addressed.  *See also changes made in response to Reviewer 2’s first comment.* |
| Trim a bit. Some sentences are long and winding. Breaking them up will keep readers from losing the thread. You don’t have to dumb it down—just give the brain a few more pauses. | We have scanned and improved the paper for readability and stylistic improvements. | *Various textual changes made throughout the paper.* |
